# Acquisition vs. Memorization Trade-Offs Are Modulated by Walking Distance and Pattern Complexity in a Large-Scale Copying Paradigm

**DOI:** 10.1371/journal.pone.0018494

**Published:** 2011-04-08

**Authors:** Gregor Hardiess, Kai Basten, Hanspeter A. Mallot

**Affiliations:** Department of Biology and Werner-Reichardt Center for Integrative Neuroscience, University of Tübingen, Tübingen, Germany; University of Chicago, United States of America

## Abstract

In a “block-copying paradigm”, subjects were required to copy a configuration of colored blocks from a model area to a distant work area, using additional blocks provided at an equally distant resource area. Experimental conditions varied between the inter-area separation (walking distance) and the complexity of the block patterns to be copied. Two major behavioral strategies were identified: in the memory-intensive strategy, subjects memorize large parts of the pattern and rebuild them without intermediate visits at the model area. In the acquisition-intensive strategy, subjects memorize one block at a time and return to the model after having placed this block. Results show that the frequency of the memory-intensive strategy is increased for larger inter-area separations (larger walking distances) and for simpler block patterns. This strategy-shift can be interpreted as the result of an optimization process or trade-off, minimizing combined, condition-dependent costs of the two strategies. Combined costs correlate with overall response time. We present evidence that for the memory-intensive strategy, costs correlate with model visit duration, while for the acquisition-intensive strategy, costs correlate with inter-area transition (i.e., walking) times.

## Introduction

Getting around in a constantly changing world relies on contributions from multiple behavioral or cognitive processes competing for common resources such as metabolic energy, information processing capacity, or processing time. The allocation of such resources to the individual processes requires some sort of “decision making” or “executive function” [1]–[4], taking into account the relative value of each choice's expected consequences, i.e. its costs and pay-offs. In this paper, we study this resource allocation process for the interaction of (or trade-off between) memorization of large amounts of information and the repeated acquisition of smaller amounts of information when acquisition involves walking between various locations. Similar interactions are common both in animal behavior and in human activities such as optimal foraging or economic decision making.

For memorization and processing of visual information, the visual working memory (WM) is an essential resource. WM can be defined as a system for maintaining and processing a certain amount of information temporarily [5], [6]. In a large body of research two general limitations of WM have been demonstrated: a temporal limitation [e.g.], [ 7]–[10] and a storage capacity limitation [e.g.], [ 11]–[13]. With respect to time, WM representations decay within several seconds when no active rehearsal processes [14] take place. Regarding storage capacity, visual WM can maintain information on approximately three to five items at a time. Additionally, these items appear to be coded in the form of integrated object representations, rather than as a collection of disconnected visual features [e.g.], [ 12], [15,16]. Visual representations in WM are maintained and updated throughout the course of a task either by using continuous, ‘just-in-time’ acquisition of environmental information, as has been shown for saccadic gaze behavior [17], or by making inferences on already existing memorized information, or both.

‘Just-in-time’ acquisition of visual information by repeated looking as opposed to keeping more information in memory [17] is a central example of strategy trade-offs minimizing certain overall costs. Such costs arise at various levels and processes including physiological costs for storing information [18], for gaze movements and redirecting attention [19], or for perceptual or attentional processing [20]. In addition, the time needed to complete a task is in itself an important cost factor, since it precludes or inhibits other relevant performances [21]. Since these costs are likely to vary with environmental and task constraints, cognitive routines are needed to balance the investment into each resource [22].

Trade-offs between gaze movements and WM use have been studied in a number of tasks which involve looking back and forth between two or more experimental areas [17], [23]–[25]. In the block-copying paradigm of Ballard et al. [17], a pattern of colored blocks is presented at a “model area”, together with additional blocks provided in a “resource area”. Subjects pick up blocks from the resource area with the computer mouse and drag them to the “workspace area” where they built a copy of the model. The more the working memory is used, the fewer fixations on the model area are required. In the comparative visual search paradigm of Hardiess et al. [24] and Pomplun et al. [25], differences between two patterns have to be detected by looking back and forth between these patterns. In both paradigms (block copying and comparative visual search), the putative costs of data acquisition are manipulated by varying the distances between the different areas and thus requiring larger or smaller gaze-shifts. In the block sorting paradigm of Droll and Hayhoe [23], blocks with different properties have to be picked up from a reservoir site and moved to one of two “conveyor belts”, depending on their property. In this paradigm, costs of memory load are manipulated by varying the predictability of the necessary information. Independent of where cost changes were applied (i.e., gaze or memory systems), all studies show an adaptation of the trade-off between gaze movement behavior and memorization processes. When the costs for gaze behavior were increased experimentally, participants shifted the balance point towards a more intense use of WM. In contrast, in the case of low stimulus predictability, participants reduced the involvement of WM and maximized the amount of gaze shifts, achieving ‘just-in-time’ processing. In summary, all investigations identified a trade-off function capable of optimizing the arising costs throughout the course of a task. Droll and Hayhoe [23] conclude that such trade-offs are an intrinsic, unconscious, pervasive, and stable aspect of human behavior.

In the above studies of the acquisition vs. memory trade-offs, acquisition behavior amounted to gaze shifts carried out by movements of the eyes and/or the head. Clearly, this kind of motor behavior is executed within small spatial scales and within very short periods of time. For example, a saccade is usually performed in less than 100 ms [26]. Together with the fixation time for extracting information (e.g., 0.4 s for fixations during making tea: [27]; 0.3 s for fixations during comparative visual search: [24]; or 0.2 s for reading: [28]) the time required for a gaze movement and thus for visual acquisition of one piece of information amounts to about one second. If acquisition is realized by gaze movements, the load that can be experimentally imposed on this side of the trade-off is therefore rather limited. It is not clear, how the strategies of resource allocation extend to acquisitive behaviors consuming much more time - in the range of several seconds.

In the present study we approach this question by replacing the gaze-shift component of the block-copying task [17], [29], [30] by actual bodily locomotion (i.e. walking) in a large room. Locomotion consumes much more time and energy than gaze movements and should therefore increase the costs for acquiring or updating information substantially. Model, resource, and workspace areas were placed at the corners of an equilateral triangle. Participants had to walk between these three operating areas to acquire new pattern information throughout the course of the copying task. In contrast to previous studies, two different types of cost were manipulated in a full factorial design: First, the costs for walking between the operating areas were varied by using two arrangements with different walking distances (“near” and “far” conditions). Second, different memorization costs were generated by using two types of block patterns, “simple” and “complex”, differing in their memory load.

The goal of the present study was to assess and quantify strategy trade-offs in a walking paradigm including manipulations in acquisition and memorization costs. With overall higher costs for locomotion compared to saccadic motor behavior, we expect a general shift of the task solving strategies towards a greater reliance on memory. Furthermore, we hypothesize that both types of cost manipulations are capable of modulating walking strategies. This modulation can be modeled as a linear optimization of combined costs.

## Methods

### Participants

48 naïve subjects volunteered to participate in this study (24 male and 24 female). Participants were under- and postgraduate students from the University of Tübingen and their ages ranged from 19 to 36 years (mean 24.6 years). Participants were paid for their participation and gave informed written consent. This research was performed in accordance with the 1964 Declaration of Helsinki and was approved by the ethical committee of the University Hospital of Tübingen.

### Material

#### Block patterns

**Figure 1:**
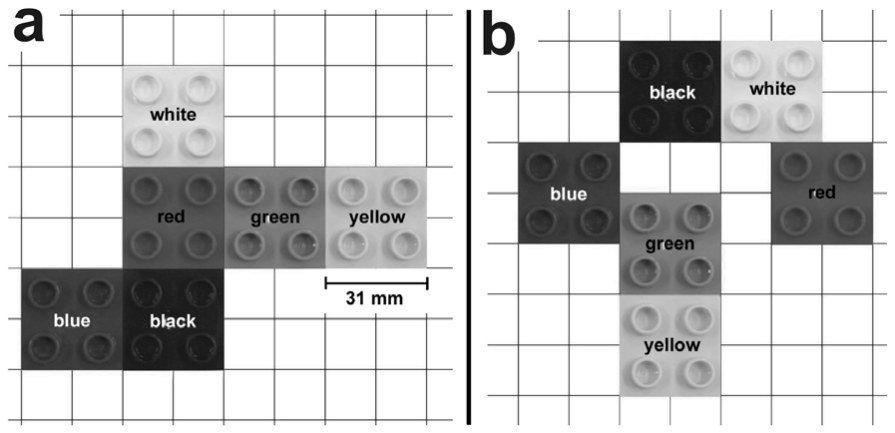
Example block-patterns. Model examples of the simple (a) and the complex (b) condition. Each pattern consisted of six quadratic LEGO® duplo® blocks (length: 32 mm, height: 24 mm) colored differently. Highlighted edges illustrate the different possibilities in which blocks could make contact with each other (black: full edge contact, white: half edge contact, and gray: diagonal block configuration, for a detailed explanation see section ‘block patterns’).

Each block pattern was composed of six quadratic LEGO® duplo® blocks of six different colors, forming a connected pattern. Blocks were placed on a grid where every block covered two by two grid cells. To control memorization demands, we varied the neighborhood rules between adjacent blocks. In “simple patterns”, adjacent blocks shared a complete edge (Figure 1a); in “complex patterns”, block adjacency could also be defined by sharing a half edge (like in a staggered brick wall) or just one corner point (diagonal neighbors, see Figure 1b). Thus, for blocks of the simple type, just one possibility or rule exists to contact another one (full edge connection), whereas for blocks of the complex patterns three of such possibilities were available (full edge, half edge, or diagonal configuration). Each complex pattern comprises one to two full edge, two to three half edge, and one to two diagonal connections. In summary, the two pattern types varied with respect to their information content (degrees of freedom), and with respect to possible chunking into salient a sub-pattern. For each type, ten different patterns were created.

#### Experimental setup

**Figure 2:**
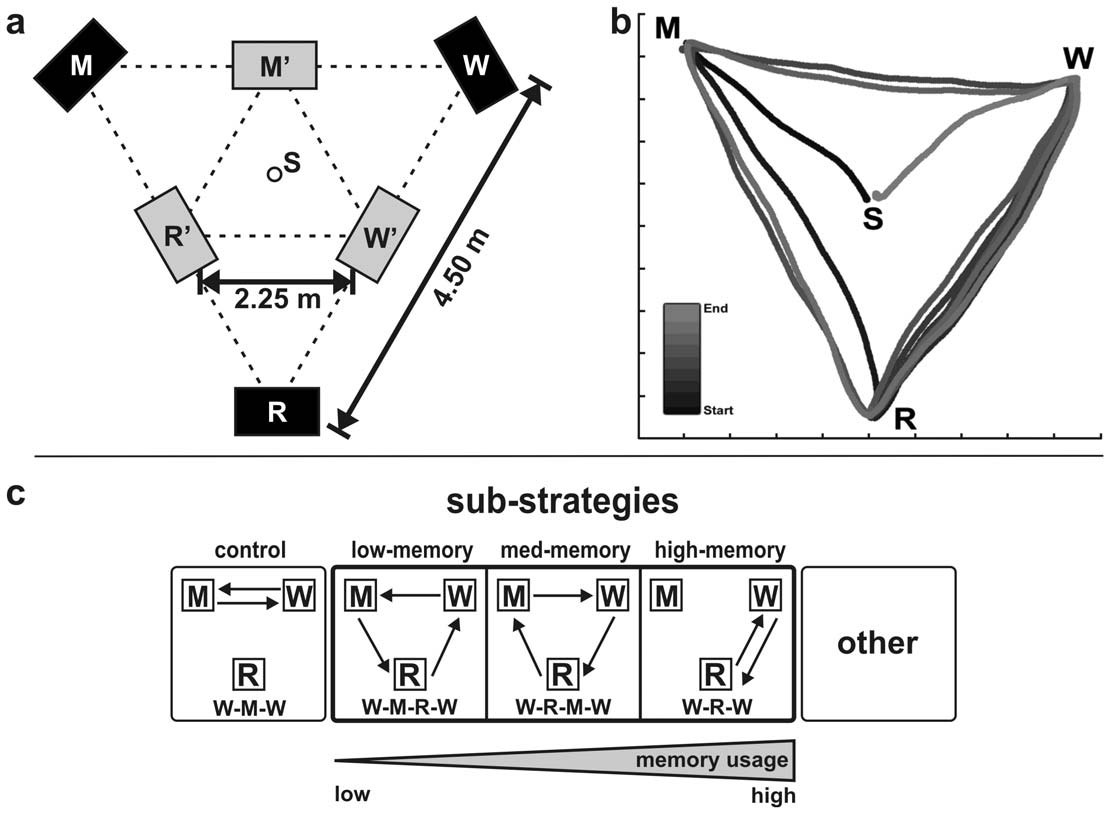
Task setup and analysis of walking trajectory. a) Scheme of the experimental setup with the spatial arrangement of the three operating areas (M: model, W: workspace, R: resource area, S: start and end point of a subjects' trajectory) for the two distance conditions (black boxes: far distance condition, gray boxes: near distance condition). b) Example of a subject's single trial trajectory in the long distance and complex pattern condition. Temporal course is coded with a gray-scale gradient. b) Relevant sub-strategies (together with their names) and their demand on WM from low to high usage. The W-M-W sub-strategy was applied as ‘control’ strategy without any block operation. ‘Other’ denotes all remaining sub-strategies which had individual frequencies of occurrence below 2% (for a detailed explanation see section ‘walking sub-strategies’).

Three separate areas arranged in the shape of an equilateral triangle were defined for model (M), resource (R), and workspace (W) operations (cf. Figure 2a). Subjects had to copy each particular block pattern presented at the model area into the workspace area by using blocks provided at the resource area. Each of the three areas consisted of a box without top cover (height 0.3 m, depth 0.22 m, width 0.3 m) placed on a 0.9 m high pedestal allowing convenient handling of the blocks. The model patterns were presented within the box at the model area. Within the box at the resource area, participants were provided with four blocks of each color for picking up. The box at the workspace was initially empty, but on the bottom a grid texture was provided allowing a more accurate alignment of blocks. The boxes were used to prevent subjects from looking at the blocks or patterns at a particular area when operating elsewhere. To vary the costs for locomotion, two different sizes of the triangular arrangement were used. In the near condition, area-to-area distance was 2.25 m while twice this distance (4.5 m) was used in the far condition (see Figure 2a).

### Position tracking

To record the walking trajectories produced by subjects while operating between the three areas (i.e., 2-dimensional body-in-space movements) an infrared light-based tracker system (ARTtrack/DTrack from A.R.T. GmbH, Weilheim, Germany) with 6 degrees of freedom was used. This device tracked a rigid target object (i.e., configuration of five light reflecting balls) that was fixed on a special helmet participants had to wear. The temporal tracking frequency of the system was 60 Hz. From the trajectories, area visits were detected using a criterion of 0.5 m. For analysis of the areas subsequently visited during a trial, subjects' body-in-space positions were evaluated (cf. Figure 2b).

### Procedure

#### Experimental groups

In a 2×2 factorial, between subject design, the 48 subjects were randomly assigned to one of four different experimental groups (12 to each group; gender was counterbalanced). Across these groups, we varied pattern complexity and locomotion distance in order to quantify the trade-off between memorization and acquisition intensive strategies. Thus, the four experimental groups include the combinations: simple/near, simple/far, complex/near, and complex/far.

#### Experimental procedure

Subjects were familiarized with the particular task operations in a pre-test where one four-block pattern had to be copied. Afterwards, each subject had to complete ten experimental trials consecutively, each with a different block pattern. Subjects started and finished each trial by standing still for about ten seconds at the central point of the triangular configuration (cf. Figure 2a and b). After each trial the copied pattern was photographed by the experimenter for later analysis of copying errors. Subsequently, all three areas were prepared for the next trial, i.e., placing a new block pattern in the model box and putting back all blocks from the workspace to the resource area. Thus, subjects found a new block pattern at the model area, a sufficient amount of blocks at the resource area, and an empty workspace area.

Subjects could visit each of the three areas in any sequence as often as necessary for replicating the current block pattern. They were instructed to do so as quickly and reliably as possible. No feedback was given to subjects during the experiment, neither about their copying performance nor about the walking strategies.

In all conditions subjects had to follow three rules throughout the copying task: i) it was forbidden to carry more than one block while walking between the areas ii) once a block was placed at the workspace area no repositioning was allowed, and iii) after placing the last block at the workspace area subjects had to go back immediately to the central point of the area configuration denoting the end of the trial.

### Data analysis

#### Copying errors

We analyzed the errors made during copying the block patterns for each of the four experimental groups. Errors were analyzed as pattern errors and block errors. Pattern errors denote the proportion of incorrectly copied patterns. Block errors denote the amount of single blocks copied at a false position or with the false color and were calculated as the proportion of the total number of blocks in the 10 patterns (n = 6 blocks×10 patterns = 60) averaged over subjects. A pattern was considered erroneous if at least one block error occurred.

#### Walking sub-strategies

The focus of the present study was to identify and characterize the trade-off between WM load and re-acquisition via locomotion. For that purpose a method was needed that assessed the extent of memory usage quantitatively. Following Ballard et al. [17] we divided the locomotion sequence of each trial into different walking sub-strategies (see Figure 2c). All sub-strategies could be classified without ambiguity.

A sub-strategy is a section of the walking sequence between two subsequent visits of the workspace area; it usually (except for the ‘control’ sub-strategy W-M-W) corresponds to the placement of one block. As an example, the sequence of visited areas …-W-R-W-M-R-W-R-M-W-… was divided into the sub-strategies W-R-W, W-M-R-W, and W-R-M-W. The W-R-W (i.e., ‘high-memory’) sub-strategy is the strategy with the highest memory involvement. Here, all required information concerning (at least) the next block (i.e., color and position) is retrieved from memory and no additional visit of the model is needed. In contrast, the sequence W-M-R-M-W (i.e., ‘just-in-time’) denotes the sub-strategy with the lowest memory involvement, where subjects walk to the model area to look for the color of the next block. After picking a suitable block from the resource area, they come back to the model area once again, presumably because they did not remember the position of that block. Thus, color is remembered during the M-R step and position during the M-W step of the sequence. In contrast to the ‘high-memory’ sub-strategy, the associated walking distance is doubled when using the ‘just-in-time’ strategy. The ‘low-memory’ sub-strategy (W-M-R-W) uses an intermediate amount of memory and path length; color has to be remembered during the M-R step, while position has to be remembered during both the M-R and R-W steps. An overview of the different sub-strategies (together with the name that is used throughout the manuscript) and the demands on memory is provided in Table 1 and Figure 2c.

**Table 1:** Sub-strategy characterization.

	sub-strategies			
memorization parameter	W-M-R-M-Wjust-in-time	W-M-R-Wlow-memory	W-R-M-Wmed-memory	W-R-Whigh-memory
# and type of block features memorized before sub-strategy	0	0	1(color)	2(color+position)
# and type of block features memorized during sub-strategy	1+1(color, position)	2(color+position)	1(position)	0
# of visits at the model during sub-strategy	2	1	1	0

Characterization of all sub-strategies used by subjects for the purpose of copying a block regarding the involvement of memory (M: model, W: workspace, and R: resource area). Each sub-strategy is given a name which is used throughout the manuscript.

Starting at the central point, the initial sub-strategy of all subjects for all trials was M-R-W. Since this sequence was a simple consequence of the task design, it was not significant for later analysis of walking strategies and thus excluded. In addition to the analysis of walking trajectories, the time subjects needed for walking and the time spent at each area was recorded. The duration for an area visit was measured from entering until leaving a catchment area defined by a radius of 0.5 meters around each operating area. The time subjects spent at the model area was separated into time for the first and subsequent visits in the course of the analysis of memorization processes.

## Results

### Task performance: Errors and overall response times

**Figure 3:**
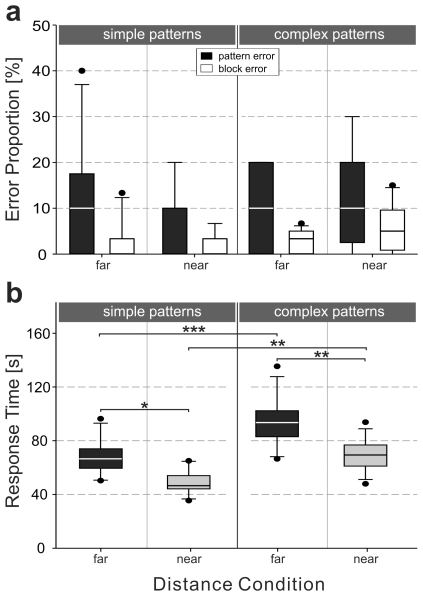
Task performance: error rate and overall response time. a) Box-Whisker plot of proportion of errors made during copying the ten simple patterns (left) and the ten complex patterns (right) for the far and the near distance conditions. Black boxes display the pattern errors: the proportion of false on all patterns (n = 10) averaged over subjects of the respective group. White boxes display the block errors: the proportion of false blocks on all blocks in all ten patterns (n = 6 blocks×10 patterns = 60) averaged over subjects of the respective group. b) Box-Whisker plot of response time to complete a single trial averaged over all subjects of the respective group for the simple (left) and complex (right) pattern situations and for the far (black boxes) and near (gray boxes) distance conditions. Statistical effects (post-hoc analyses) are presented for each pattern complexity/distance combination (^★^p<.05; ^★★^p<.01; ^★★★^ p<.001).

Task performance was quantified by the number of pattern errors and block errors. In all conditions participants showed a high level of performance, i.e., on average 9 out of 10 patterns were copied correctly (Figure 3a). Furthermore, only about two to three blocks out of all 60 blocks were copied at a false position. Statistical analysis showed no influence of distance condition or pattern complexity on pattern errors (Kruskal-Wallis-Test: χ^2^ = 1.4, p = .71) nor on block errors (Kruskal-Wallis-Test: χ^2^ = 3.78, p = .29).

Response time was analyzed in terms of the overall time participants needed to finish a single trial (Figure 3b). For an analysis of durations of model visits, see section ‘memorization and model usage’ below. Regarding overall time, a two-factorial ANOVA with pattern complexity (complex vs. simple) and distance condition (far vs. near) as factors was conducted. We found significant main effects of pattern complexity (F(1,44) = 34.59, MSE = 173.54, p<.001, η_p_^2^ = .44) and the distance condition (F(1,44) = 56.81, MSE = 173.54, p<.001, η_p_^2^ = .56). We found no interaction between these two factors (F(1,44) = 1.17, MSE = 173.54, p = .28). Independent of pattern complexity, subjects needed significantly more time to reproduce the patterns in the far as compared to the near distance condition (Figure 3b). Additionally, compared to the simple pattern condition, trial duration for the complex patterns was significantly increased in both distance conditions.

### Locomotion strategies

As described in the methods section (cf. ‘walking sub-strategies’), trajectories of each trial were analyzed by means of a number of sub-strategies indicating various amounts of memory usage. Separately for each experimental group the frequency of occurrence of these sub-strategies was evaluated. A summary of sub-strategies together with their demand on WM is given in Figure 2c and Table 1. Overall, two predominantly used sub-strategies (i.e., ‘high-memory’ and ‘low-memory’) and several sub-strategies with generally low occurrence (below 5%) were found in all four experimental groups.

**Figure 4:**
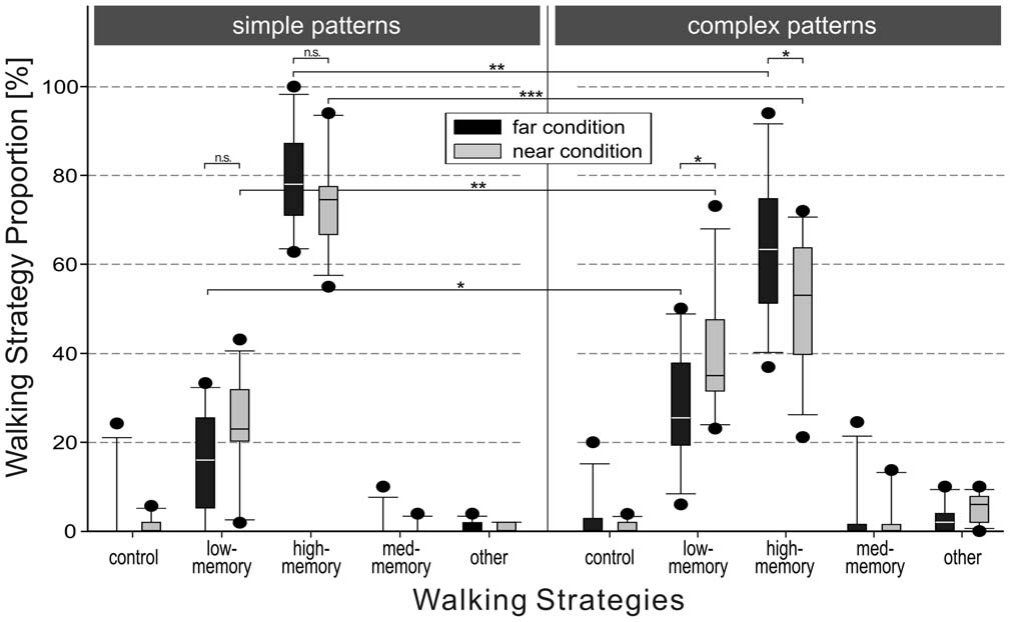
Proportion of walking sub-strategies. Box-Whisker plot of proportion of walking sub-strategies used by subjects during copying the simple patterns (left) and the complex patterns (right) averaged over all subjects of the respective group. Black boxes display the frequencies of walking sub-strategies for the far distance condition and gray boxes these for the near distance condition. Post-hoc analyses are calculated for ‘low-memory’ and ‘high-memory’ referring the proportion of walking sub-strategies between far and near and simple and complex pattern conditions (^★^p<.05; ^★★^p<.01; ^★★★^ p<.001; n.s. not significant). The characteristics of all individual sub-strategies are explained in detail in the results chapter (see section ‘walking sub-strategies’).

The sub-strategies with low frequencies of occurrence (see Figure 4) were ‘control’ (means between .96 and 3.15%), ‘med-memory’ (means between .5 and 3.38%), and ‘other’ (means between .92 and 4.93%). The category of ‘other’ contains the sum of all remaining walking strategies with individual frequencies of occurrence below 2% on average. Interestingly, the sub-strategy with the lowest memory involvement at all, i.e., ‘just-in-time’, was part of the ‘other’ category; it was applied only in the complex/near and the complex/far group with means of 1.45% and .16%, respectively. We never found this sub-strategy in any of the near distance trials. The ‘control’ sub-strategy was applied as the control strategy without any block operation.

The two main (i.e., predominantly used) sub-strategies were ‘high-memory’ (WRW) and ‘low-memory’ (WMRW, see Figure 4). The ‘high-memory’ sub-strategy was applied if subjects could rely on memories of both color and position of (at least) the next block. No model visit was required with this sub-strategy. In contrast, if neither color nor positional information of the next block was available from memory the ‘low-memory’ sub-strategy was applied. In this case, subjects had to visit the model for memorizing both block features before picking up and placing the block.

Statistical analysis reveals an influence of both, distance and pattern complexity on the frequency of occurrence of the two main sub-strategies. By calculating a two-factorial ANOVA for the proportion of ‘high-memory’, we identified main effects of pattern complexity (simple vs. complex: F(1,44) = 22.15, MSE = 187.42, p<.001, η_p_^2^ = .33) and distance (far vs. near: F(1,44) = 4.9, MSE = 187.42, p<.05, η_p_^2^ = .1). Thus, with an increase in distance and a decrease of pattern complexity the trade-off between acquisition and memory is shifted towards the memory-intensive sub-strategy ‘high-memory’. This shift of the trade-off is also reflected in the other main sub-strategy ‘low-memory’. Here, the influence of both factors was inverted (complexity: F(1,44) = 15.38, MSE = 160.16, p<.001, η_p_^2^ = .26; distance: F(1,44) = 7.33, MSE = 160.16, p<.01, η_p_^2^ = .14). A decrease in distance and an increase in pattern complexity induced a shift of the trade-off between acquisition and memory towards the ‘low-memory’ sub-strategy. For both main sub-strategies no significant interaction between the two factors (distance and complexity) was found (‘high-memory’: F(1,44) = .75, MSE = 187.42, p = .39; ‘low-memory’: F(1,44) = .44, MSE = 160.16, p = .51). However, based on the estimated parameters of the linear model of the ANOVA on the proportion of ‘high-memory’ (goodness of fit = R_adj_^2^ = .35) a difference in distance condition depending on model complexity was apparent, indicating an ordinal interaction.

### Memorization and model usage

In order to assess the degree to which the model is used in the various conditions, we analysed the duration and number of model visits and the number of blocks processed after the initial model visit of each trial.

**Figure 5:**
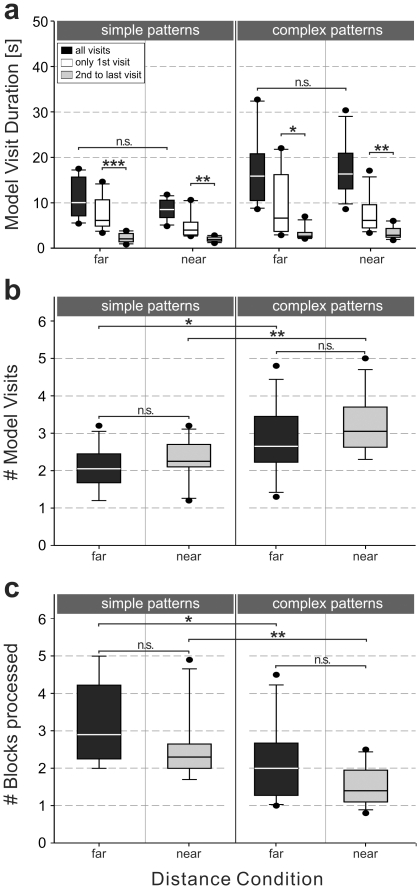
Model operations. a) Box-Whisker plot of time subjects spent to visit the model area averaged over all subjects of the respective group for the simple (left) and complex (right) pattern situations and for the far and near distance conditions. Black boxes display the total time, subjects spent at the model. White boxes display the duration of the first model visit. Gray boxes display the average duration of individual subsequent model visits. Statistical effects (t-test) are calculated between first model visit and second to last model visit times for each pattern complexity/distance combination. Post-hoc analyses are calculated between the distance conditions for each complexity. b) Box-Whisker plot of the number of model visits per trail averaged over all subjects of the respective group for the simple (left) and complex (right) pattern situations and for the far (black boxes) and near (gray boxes) distance conditions. Statistical effects (post-hoc analyses) are presented for each pattern complexity/distance combination. c) Box-Whisker plot of the number of blocks processed after the initial model visit per trail (i.e., number of consecutive ‘high-memory’ cycles after the initial model visit) averaged over all subjects of the respective group for the simple (left) and complex (right) pattern situations and for the far (black boxes) and near (gray boxes) distance conditions. Statistical effects (post-hoc analyses) are presented for each pattern complexity/distance combination. (^★^p<.05; ^★★^p<.01; ^★★★^ p<.001; n.s. not significant).

The total time for all visits subjects spent at the model (Figure 5a) depended on pattern complexity but not on distance (two-factorial ANOVA, complexity: F(1,44) = 20.1, MSE = 39.92, p<.001, η_p_^2^ = .31; distance: F(1,44) = .9, MSE = 39.92, p = .35). Within each experimental condition, subjects spent significantly more time for the first model visit compared to subsequent model visits (Figure 5a). Similar to the results for the overall response time, the time for initial memorization (i.e., first model visit) was also found to increase with longer distance and higher pattern complexity (Figure 5a). However, for the initial memorization times, dependence on conditions did not reach significance in a two-factorial ANOVA with pattern complexity (complex vs. simple) and distance condition (far vs. near) as factors (complexity: F(1,44) = 2.8, MSE = 37.02, p = .1; distance: F(1,44) = 3.41, MSE = 37.02, p = .07). Since no significant differences were found for subsequent model visits within each experimental group all subsequent time values were averaged for each group. Regarding time for subsequent model visits, a two-factorial ANOVA with pattern complexity and distance condition as factors was conducted. We found only a significant main effect of pattern complexity (F(1,44) = 12.881, MSE = 1.297, p<.01, η_p_^2^ = .23). No significant influence of the distance (F(1,44) = .012, MSE = 1.297, p = .91) was found.

To estimate the amount of memory subjects allocated in the different experimental conditions we analyzed the number of model visits per trial and the number of consecutive ‘high-memory’ cycles after the initial model visit (Figure 5b and c). Initial and consecutive ‘high-memory’ cycles were chosen for two reasons: i) the initial ‘high-memory’ cycles include only knowledge obtained through a single memorization process (first model visit) without pre-knowledge of the pattern and ii) consecutive ‘high-memory’ cycles exclude intermediate memory refresh.

The number of model visits per trial was dependent only by pattern complexity but not by the distance (two-factorial ANOVA, complexity: F(1,44) = 13.86, MSE = .55, p<.001, η_p_^2^ = .24; distance: F(1,44) = 2.14, MSE = .55, p = .15). Subjects visited the model for memory refresh more often in the complex conditions (means: 2.77 for the far and 3.19 for near distance) than for simple patterns (means: 2.08 for the far and 2.28 for near distance).

We found an overall higher number of consecutive ‘high-memory’ cycles in the simple pattern conditions (means: 3.2 for the far and 2.57 for near distance) than in the complex pattern conditions (means: 2.18 for the far and 1.54 for near distance). Within pattern complexity, also an increase of memorization was found with an increase of the distance. The two-factorial ANOVA with pattern complexity (complex vs. simple) and distance condition (far vs. near) as factors showed significant main effects of pattern complexity (F(1,44) = 14.03, MSE = .89, p<.001, η_p_^2^ = .24) and distance condition (F(1,44) = 5.33, MSE = .89, p<.05, η_p_^2^ = .11). No significant interaction between these two factors was found.

In a trial by trial analysis of blocks processed after the initial model visit, we found moderate correlations between the duration of the initial model visit and the number of consecutive ‘high-memory’ cycles. Correlations reached significance for all conditions (simple/far: Rho-S = .23, p<0.05; simple/near: Rho-S = .51, p<0.01; complex/far: Rho-S = .6, p<0.01; complex/near: Rho-S = .34, p<0.01).

### Trade-off stability

**Figure 6:**
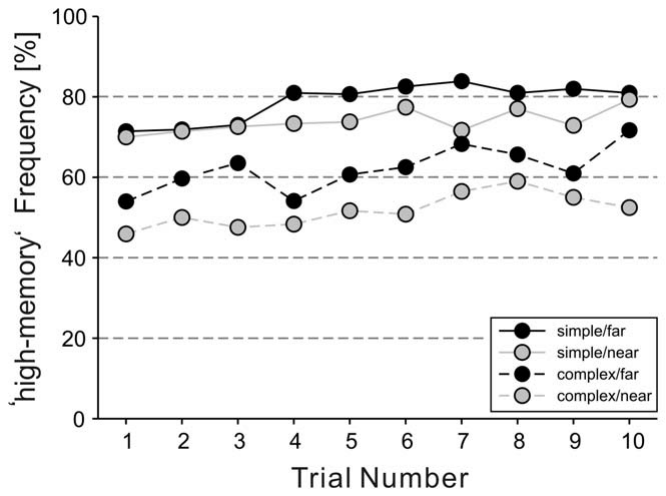
Sub-strategy stability over trials. Occurrence of the main sub-strategy ‘high-memory’ as a function of trial number. The frequency of the sub-strategy was averaged over all subjects within the respective group and is plotted separately for each experimental group.

The influence of trial order on the occurrence of used sub-strategies was analyzed by comparing the frequencies of sub-strategies per trial within each experimental group. No influence of the trial order on any of the walking sub-strategies could be found. Figure 6 illustrates this stability of sub-strategy usage within each experimental group for the main sub-strategy ‘high-memory’.

## Discussion

Working memory (WM) supports many higher cognitive functions by maintaining representations of a limited number of items, and by selecting and attending to those representations which are most relevant for the current task. Memory items include multiple types of information, such as verbal or visuospatial, as well as task rules and are represented in WM through sustained patterns of neural activity [31]. Executive decisions or choices are needed between all possible and competing courses of actions based on the relative value of their expected consequences [2]–[5]. The allocation of resources to the competing strategies or actions requires trade-off decisions which will show in the preference for one or another strategy or action, and in the dependence of such trade-offs on task constraints. [22], [32].

The main objective of the present study was to investigate if such a trade-off, already described for the balancing of WM and gaze movements [17], [23]–[25], also exists for locomotion behavior, i.e., actions that demand larger time frames (seconds) and distance scales (meters). If such a trade-off exists, variation of memory load and required walking distance should affect the behavioral strategies employed by the subjects. To prove this hypothesis, the block-copying task introduced by Ballard et al. [17] was adapted to fit the needs of our walking paradigm. Additionally, pattern complexity was added as a second dependent variable to investigate the relative weights of locomotion and memory load.

### Strategy trade-off

The main result of this paper is that alternative behavioral strategies, which can be used to achieve the same goal, are used to various extents if task parameters are varied. In our block-copying task, the main behavioral strategies are i) initial acquisition of large amounts of information and subsequent operation from memory (“memory-intensive strategy”), and ii) the repeated acquisition or re-acquisition of smaller pieces of data and subsequent processing of these individual packages (“acquisition-intensive strategy”). The memory-intensive strategy is realized by long initial model visits and high proportions of the ‘high-memory’ sub-strategy. The acquisition-intensive strategy, in contrast, uses relatively short initial model visits and a high proportion of the ‘low-memory’ sub-strategy.

For more complex patterns, the use of the memory-intensive strategy is reduced, while more re-acquisition steps are performed. A similar but weaker effect was found for walking distance: if walking of larger distances is required, the frequency of the memory-intensive strategy increases.

The observed pattern of the dependence of sub-strategy usage (Figure 4) on conditions is evidence for a functional trade-off balancing the relative costs of WM involvement (memory-intensive strategy) and locomotion behavior (acquisition-intensive strategy). The nature of these costs will be discussed in more detail below. Here we note that the variations in the costs of each strategy induced by varying pattern complexity seem to be larger than the variation induced by walking distance. Thus, the condition-dependent strategy shift for simple vs. complex pattern is more pronounced than for the near vs. far conditions.

As compared to the findings of Ballard et al. [17], who used gaze shifts rather than locomotion, we find a much smaller frequency of the ‘just-in-time’ sub-strategy W-M-R-M-W. This sub-strategy was the main strategy with a frequency of about 35% in the gaze-study, whereas our results show a predominant use of the ‘high-memory’ sub-strategy in all experimental conditions (between 50% and 80%) and only a negligible proportion of the ‘just-in-time’ processing strategy (less than 2%). We conclude that for our task parameters (walking distance and pattern complexity), the trade-off operated on an overall higher memory level, whereas in the case of the gaze movement experiment, the trade-off seems to operate on a lower memory level. This general shift towards higher memory involvement was most likely induced by the overall higher costs for acquisition; pattern complexity had a stronger influence on the selection of sub-strategies at this high memory level.

### Processing time and memory operations

As measures of memory involvement in a given strategy we analyzed the duration and number of model visits (i.e. the time spent with the encoding of information) and the number of blocks processed (i.e., the information taken from memory while copying), see Figure 5. As a consistent result, we found that for the simple pattern condition (both in the near and far case), model visit duration is shorter, the number of model visits is smaller, and the number of blocks processed after a model visit is larger than in the complex pattern condition. This indicates that the complex pattern condition (as compared to the simple one) requires more and longer model visits to build up memory. The memorized information, however, suffices only for the placement of a smaller number of blocks. Also, when comparing near and far conditions, the number of blocks processed after a model visit is larger in the far condition, indicating that more memory has been stored.

These findings support the idea that longer and more frequent model visits lead to the build-up of extended memory which in turn is available for the processing of blocks. This idea can be tested directly by calculating within each condition trial-by-trial correlations between the duration of the first model visit and the number of blocks processed consecutively. Here we did indeed find moderate but significant correlations.

The first model visit was significantly longer than the second or any later visit, indicating that it plays a special role. Conceivably, the subject could use this first visit to built up a memory of some global features of the pattern which is not necessarily used for immediate block positioning but may be useful for later information intake. Examples of such features are chunks or templates known to reduce working memory load [33], [34].

Simple and complex patterns require different amounts of storage capacity. A simple estimate of this capacity can be derived from the following consideration: Suppose the first block of a simple pattern is placed. For the next block, there are four possible positions observing the neighborhood rules described in the methods section (Figure 1). Ignoring effects of boundaries and mutual intersection of individual block positions, the number of possible six-block patterns will be about 4^5^ = 2^10^, corresponding to an information content of 10 bits per pattern. A similar calculation for the complex pattern, where 16 positions of the second block are possible, yields 16^5^ = 2^20^ possible patterns corresponding to an information content of 20 bits per pattern. If we assume that the same total memory capacity is used for both cases, we should expect that the number of blocks processed per model visit in the simple conditions is about twice the number processed in the complex conditions. As can be seen from Figure 5c, this ratio is about 1.5 to 1 in our data. Clearly, the above calculation suffers from a number of shortcomings which may cause the observed deviation. First, the actual number of patterns is smaller than assumed, since the possible positions for block placement are constraint by previously placed blocks. Second, if the assumed trade-off actually takes place, the memory capacity allocated to the task should be larger in the complex condition, predicting ratios below 2 to 1. Third, memory capacity needed for the storage of a pattern will depend on chunking or the possibility of recognizing templates in the pattern. Simple block configurations are more likely to comprise familiar sub-patterns such as letter shapes (e.g., ‘I’ or ‘L’) or other geometric figures that facilitate memorization. Chunking processes serve to bind isolated pieces of information together to form a meaningful combination (block positions and sub-patterns) that can be associated with previously stored long-term memories. However, we found that subjects processed between 1.54 and 3.2 blocks in a row (i.e., without model visits in between), a rate that is below the range of the generally assumed WM capacity of 3 to 7 items [e.g.], [ 12], [16,35]. Thus, it seems that one block in our task is represented by more than one of the WM items as discussed in the gaze-shift literature cited above. Such an increased amount of WM demand could be caused by the elongated maintenance of memory [e.g., 36] required in our large-scale walking paradigm but not in gaze-shift paradigms. Moreover, temporal forgetting of memorized material is caused by memory decay [37] and processes of interference [38].

### Costs and optimization

The trade-off idea states that behavioral strategies are selected so as to minimize certain task and condition dependent costs. Generally two types of costs are considered: i) processing time and ii) energy consumption or other, non-temporal measures of cognitive effort. The soft constraints hypothesis Gray et al. [21] suggests that on the memory side “the only factors that matter are the time required to encode, the time required to retrieve an item from memory, and the probability that an encoded item can be retrieved (i.e., is not forgotten) when needed”. That is, the soft constraints hypothesis presupposes a control system selecting sequences of routines (sub-strategies) that tend to minimize performance costs measured in time for processing (i.e., temporal cost-benefit trade-offs). At the same time, the amount of memory used may gradually change, in relation to the costs incurred by acquisition-intensive strategies. Alternatively, it has been suggested that behavioral decisions are always made so as to minimize WM allocation [23], [39], [40] even when the costs of information access (as measured by time) for ‘just-in-time’ (perceptual-motor) strategies are greater than the costs for memory strategies [41]. Since WM capacity is limited, the control system is biased to assign work to the perceptual-motor system [42]. For block-copying, Ballard et al. [41] reported that participants preferred a ‘just-in-time’ (i.e., low WM allocation) strategy that took 3 s to execute over the more memory-intensive strategy that took 1.5 s to execute. Hence time was not the factor determining WM processes in that task. We suggest that the reason why the ‘just-in-time’ strategy is not used in our experiment is the higher cost of inter area transitions associated with physical walking as compared to mere gaze-shifts. In any case, the data reported here seem to support a soft constraint scheme in which memory costs can be “traded” for acquisition costs. For further experimental evidence supporting the soft constraints and minimum memory hypotheses, see [24] and [23].

**Figure 7:**
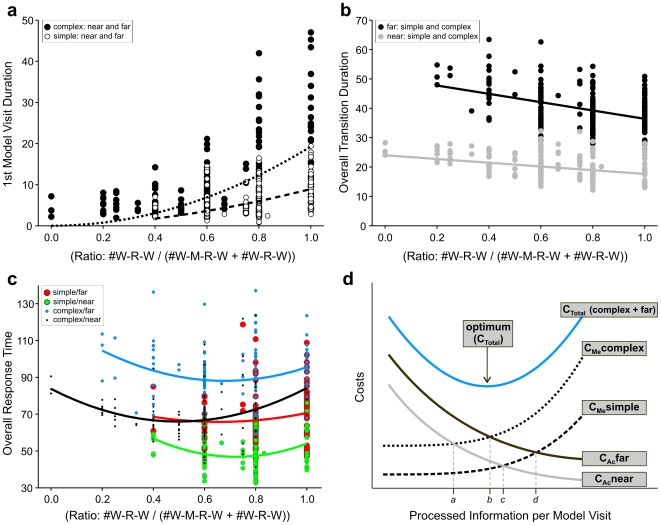
Linear cost optimization. Experimental data for a) costs for memorization (i.e., duration of 1st model visit) with power function fits for simple (dashed line) and complex (dotted line) patterns, b) costs for acquisition (i.e., overall time for transitions) with linear regression lines for near (gray) and far conditions (black), and c) total time costs (i.e., overall response time; regressions indicate quadratic functions). All data are shown as a function of the ratio between ‘high-memory’ and ‘low-memory’ sub-strategies. d) Model: Total costs are divided in costs for memorization (C**_Me_**) and acquisition (C**_Ac_**). If more information is processed at each model visit (i.e., if the task is solved with fewer visits), memory costs increase while acquisition costs decrease. These individual costs vary also with the experimental conditions for walking distance (near and far) and pattern complexity (simple and complex). Total costs for the complex/far condition are depicted as the sum of the according individual cost curves (blue line), leading to an optimum of processed information per model visit at point *b*. The location of each optimum for the four experimental groups is indicated with *a–d*.

As revealed by the overall response times (Figure 3b), harder tasks (i.e., the far and complex conditions) require longer overall time. It therefore seems likely that time does play a role as a cost factor in our experiment. To further analyze this hypothesis, we evaluated three timing parameters, i) the initial model visit duration, ii) overall walking time, and iii) overall response time. Within each condition, we analyzed the frequency of the various sub-strategies per trial. Note that these data do not appear in Figure 4 which shows only the frequencies averaged over all trials and subjects within each condition. Next we analyzed the dependence of the three timing parameters on the sub-strategy frequencies per trial. Strategy shift as depicted in Figure 4 is mostly between the sub-strategies ‘high-memory’ (W-R-W) and ‘low-memory’ (W-M-R-W). We therefore expressed the strategy shift by the ratio of sub-strategy usage, (#W-R-W/(#W-R-W+#W-M-R-W)) for each trial. Figure 7a–c shows the timing parameters as a function of this sub-strategy ratio. The duration of the initial model visit (Figure 7a) increases with the proportion of the ‘high-memory’ sub-strategy. It may thus be considered a cost factor associated with memorization, favoring the ‘low-memory’ sub-strategy. The curves show power functions fitted to all trials in the simple and complex conditions (lumping together near and far). Conversely, overall walking time (Figure 7b) increases with the proportion of the ‘low-memory’ sub-strategy. It may thus be considered a cost factor associated with acquisition, favoring the ‘high-memory’ sub-strategy. The curves show linear regression lines fitted to all trials in the near and far conditions (lumping together simple and complex). The overall response time (Figure 7c) shows a U-shaped dependence on sub-strategy ratio. The curves are second order polynomials fitted to all trials of each of the four conditions. The minima of the U-curves for the four conditions appear in the order complex/near<complex/far<simple/near≈complex/near, which is consistent with the actual strategy usage shown in Figure 4.

Figure 7d shows the overall idea of trade-offs generated by the minimization of combined costs (soft constraints). The individual components, i.e. acquisition costs and memorization costs follow convex functions, either decreasing or increasing, whose vertical position depends on the experimental condition. Acquisition costs are assumed to depend only on walking distance and memorization costs are assumed to depend only on pattern complexity. The blue curve shows the sum of the acquisition costs in the far conditions and the memorization costs in the complex conditions. Its minimum corresponds to the sub-strategy ratio optimizing overall response time in the complex/far condition. Models for the other U-curves are obtained by summing the respective individual cost curves, but are not shown in the figure. Their minima occur roughly at the intersections of the individual cost curves and are marked by letters *a*–*d* in the figure. The model is in good general agreement with the data shown in Figure 7a–c.

In this analysis, the individual cost functions are assumed to be stable and known by the trade-off controller. This is in line with the trade-off stability reported in Figure 6: subjects use the same sub-strategies throughout the course of the experiment without need to adjust to the experienced costs.

In summary, the analysis presented in Figure 7 supports the idea that time is a correlate of overall costs and the trade-off results from the optimization of combined soft constraints (continuous cost functions in Figure 7a and b) for which again temporal correlates can be given. We cannot exclude the possibility that non-temporal costs (e.g., energy consumption, distraction of WM from other important tasks, memory decay) play a role, since they are likely correlated with temporal parameters.
